# The psychopathology of NMDAR-antibody encephalitis in adults: a systematic review and phenotypic analysis of individual patient data

**DOI:** 10.1016/S2215-0366(19)30001-X

**Published:** 2019-03

**Authors:** Adam Al-Diwani, Adam Handel, Leigh Townsend, Thomas Pollak, M Isabel Leite, Paul J Harrison, Belinda R Lennox, David Okai, Sanjay G Manohar, Sarosh R Irani

**Affiliations:** a Oxford Autoimmune Neurology Group, Nuffield Department of Clinical Neurosciences, University of Oxford, Oxford, United Kingdom; b Department of Psychiatry, University of Oxford, Oxford, United Kingdom; c Department of Psychosis Studies, Institute of Psychiatry, Psychology and Neuroscience, King's Health Partners, London, UK; d Department of Neurology, John Radcliffe Hospital, Oxford University Hospitals NHS Foundation Trust, Oxford, UK; e Department of Psychological Medicine, John Radcliffe Hospital, Oxford University Hospitals NHS Foundation Trust, Oxford, UK; f Oxford Health NHS Foundation Trust, Warneford Hospital, Oxford, UK

## Abstract

**Background:**

Early immunotherapy administration improves outcomes in patients with N-methyl-D-aspartate receptor (NMDAR)-antibody encephalitis. As most patients with NMDAR-antibody encephalitis present to psychiatrists, the psychopathology of NMDAR-antibody encephalitis needs to be clearly defined to encourage accurate clinical identification and prompt treatment.

**Methods:**

For this systematic review, we searched PubMed for all studies published in English between Jan 1, 2005, and Oct 7, 2017, to identify individually reported adult patients (≥18 years) who satisfied consensus criteria for definite NMDAR-antibody encephalitis. After generating a list of 50 fine-grained, lower-level features, we extracted psychopathological data in addition to demographic and aetiological data. The lower-level features were later ordered within higher-level categories. As a means of quality control, we filtered the data according to proxy markers of psychiatric involvement in their description. Subsequently, we compared lower-level features from individual patient data with operationalised psychiatric syndromes using a constrained combination approach and principal component analysis, and did a network analysis to explore the inter-relationships between multiple lower-level features. The review protocol was prospectively registered with PROSPERO, number CRD42017068981.

**Findings:**

Of 1096 records identified in PubMed, 333 satisfied inclusion criteria and described 1100 patients in total with NMDAR-antibody encephalitis. The psychopathology of 505 (46%) patients with reported psychiatric symptoms was described in more detailed terms than only psychiatric or behavioural. 464 (91%) of the 505 patients were from papers in which patient data were reported individually. The remainder of the analyses focused exclusively on these 464 patients. Median age was 27 years (IQR 22–34), 368 (79%) of 464 patients were female and in 147 (32%), NMDAR-antibody encephalitis was associated with ovarian teratoma. The five higher-level categories into which the 464 patients most frequently grouped were behaviour (316 [68%]), psychosis (310 [67%]), mood (219 [47%]), catatonia (137 [30%]), and sleep disturbance (97 [21%]). The overall pattern of lower-level features was statistically stable across subgroups classified by age, sex, pregnancy association, presence of ovarian teratoma, prior herpes simplex virus encephalitis, and isolated psychiatric presentations (two-way ANOVA p=0·6–0·9). Constrained combination and principal component analyses found that mixtures of mood and psychosis syndromes fit each patient better than any single diagnosis alone, particularly for the patients in the psychiatric-described subgroup (mean ΔAkaike information criterion −0·04 in non-psychiatric-described subgroup *vs* 0·61 in psychiatric-described subgroup). The overlapping nature of the higher-level features was also enriched upon analysis of the psychiatric-described data (221 [67%] of 329 overlaps in non-psychiatric-described subgroup *vs* 96 [81%] of 118 overlaps in psychiatric-described subgroup, p=0·0052). Network analysis confirmed that the features were closely related and consistent between individual patients; the psychiatric-described subgroup had a markedly high and narrow range of closeness centralities (92% above 0·93 in psychiatric-described subgroup *vs* 51% above 0·93 in the non-psychiatric group).

**Interpretation:**

The distinctive aspect of NMDAR-antibody encephalitis psychopathology is complexity; core aspects of mood and psychotic disorders consistently coexist within individual patients. Alongside the predominant young female demographic, these psychopathological features could help psychiatrists identify patients who would benefit from cerebrospinal fluid testing and immunotherapies. Well-controlled prospective studies with bespoke inventories are needed to advance this clinically grounded approach.

**Funding:**

Wellcome Trust, NIHR Oxford Biomedical Research Centre, NIHR Oxford Health Biomedical Research Centre, British Medical Association Foundation for Medical Research.

**Research in context**

**Evidence before this study**

Psychiatrists are increasingly aware that NMDAR-antibody encephalitis, a potentially fatal yet highly treatable disease, often presents with rapid onset to mental health services. Widespread screening based on serum testing alone might predispose to misdiagnosis and iatrogenic harm in patients with clinically irrelevant seropositivity. By contrast, because cerebrospinal fluid is considered to give a definitive result, the pivotal clinical decision is whether the psychiatric presentation justifies a lumbar puncture. As lumbar punctures are invasive and rarely requested within mental health services, defining clinical characteristics of the psychiatric presentation of NMDAR-antibody encephalitis is central to rational patient selection, but the nature of this pattern has not been systematically studied. We searched PubMed for all studies published in English between Jan 1, 2005, and Oct 7, 2017, using the search terms ((anti-N-methyl-d-aspartate OR NMDA receptor OR NMDAR OR NMDAR-antibody OR anti-NMDA OR anti-NMDAR OR NMDA OR NMDA-antibody) AND (encephalitis OR autoimmune encephalitis)) but found no systematic extraction and analysis of the available data concerning the mental state of patients with NMDAR-antibody encephalitis. During the final stages of our study, one systematic review was published.

**Added value of this study**

We created a list of features that we would look for in a psychiatric interview as well as those we thought patients with NMDAR-antibody encephalitis might have on the basis of our pre-existing clinical experience of the disease. We refined the list of features until we had 50 reported lower-level features, and then used these to extract psychopathological concepts from our systematic review of published reports of NMDAR-antibody encephalitis. We analysed a large cohort of individual patient data, and found a complex pattern that crossed multiple domains of psychopathology. With computational modelling, we observed that mixed mood-psychosis syndromes fit each patient better than any single diagnosis alone. The frequency of psychopathological features was stable across demographic and aetiological variables, and its network features were highly coherent, particularly after enrichment for reports reflecting greater expertise in descriptive psychopathology.

**Implications of all the available evidence**

NMDAR-antibody encephalitis might best be described as a mixed mood-psychosis syndrome. Given the transdiagnostic psychopathology, multistage neurological features, established subacute onset, and predominant young female demographic, a clear clinical index of suspicion for the earliest stages of this treatable condition can now be developed. The selective and clinically driven approach supported by our findings provides evidence against widespread unselected serum screening and might facilitate early diagnosis of NMDAR-antibody encephalitis. To evaluate the specificity of such clinical markers and their positive predictive value, construct-appropriate standardised scales should be measured in cohorts of patients with primary psychiatric disorders and compared with cohorts of patients with NMDAR-antibody encephalitis. Early involvement by psychiatrists alongside neurology colleagues is crucial not only in clinical practice but also in the design of future multicentre studies. Our observations should prompt an urgent conversation about how, in the appropriate clinical setting, mental health services can best deliver lumbar punctures to appropriately selected subgroups of patients with acute severe mental illness.

## Introduction

N-methyl-D-aspartate receptor (NMDAR)-antibody encephalitis is an autoantibody-mediated disease that typically presents with psychiatric features before progressing to seizures, a complex movement disorder, autonomic dysfunction, and hypoventilation.^1^ Two identified triggers are an underlying ovarian teratoma and herpes simplex virus encephalitis.^1^, ^2^, ^3^, ^4^

Observational evidence consistently associates earlier administration of immunotherapies with improved outcomes.^5^, ^6^ Conversely, delays in distinguishing this disorder from a primary psychiatric syndrome can have serious consequences, with a mortality of up to 25% in patients receiving limited or delayed immunotherapy.^5^ Additionally, certain psychotropic medications can worsen symptoms and patients with NMDAR-antibody encephalitis have a lower threshold for neuroleptic malignant syndrome.^7^

Psychiatric units are poorly suited to managing complications such as seizures and autonomic instability,^8^ and they very rarely have access to lumbar punctures, an important consideration given that cerebrospinal fluid (CSF) testing is required to diagnose NMDAR-antibody encephalitis definitively.^5^, ^9^ In clinical practice, an organic diagnosis is often considered only when unequivocal neurological features are present.^10^, ^11^, ^12^ Yet, around 95% of adult patients with NMDAR-antibody encephalitis have a psychiatric clinical picture, usually at illness onset, and some have protracted isolated psychiatric presentations.^1^, ^5^, ^6^, ^10^, ^11^, ^12^, ^13^, ^14^

Therefore, the development of a clinical approach to triaging patients with new-onset mental illness for NMDAR-antibody encephalitis is required. We aimed to characterise the psychopathology in definite adult cases to focus consideration of timely CSF testing and early immunotherapy in a clinically defined population.

## Methods

### Search strategy and selection criteria

This systematic review adhered to the Preferred Reporting Items for Systematic reviews and Meta-Analysis (PRISMA) guidelines. We searched PubMed for all studies published in English between Jan 1, 2005, and Oct 7, 2017 using the search terms ((anti-N-methyl-d-aspartate OR NMDA receptor OR NMDAR OR NMDAR-antibody OR anti-NMDA OR anti-NMDAR OR NMDA OR NMDA-antibody) AND (encephalitis OR autoimmune encephalitis)). Bibliographies of included studies were screened for additional reports.

To identify all adult cases of NMDAR-antibody encephalitis, two reviewers (AAD and LT) independently screened titles and abstracts of the identified studies. Studies were included if patients had a definite diagnosis of NMDAR-antibody encephalitis based on consensus criteria^9^ and were at least 18 years old (to reflect clinical practice and exclude younger patients, who tend to have an early and dominant movement disorder phenotype),^5^ and if sufficient clinical detail was available to permit data extraction.

### Data analysis

For data extraction, first we generated a comprehensive list of features likely to be detected in a typical psychiatric interview. These features included core aspects of common psychiatric syndromes—for example, delusions, hallucinations, and depressed mood. After reviewing the first 100 reports, we modified the list to reflect emergent unforeseen features (eg, wandering aimlessly, incongruent laughter-crying) and remove redundant features (eg, purging, eating sweet foods). This resulted in 50 lower-level, relatively fine-grained features that were grouped post hoc into eight higher-level, clinically pragmatic categories. Data extraction was restarted with these 50 features and this list was found to be adequate in capturing psychiatric descriptions from all reports. Based on reported details, the final dataset contained both categorisation of reported lower-level features into higher-level features, and instances in which only higher-level features were reported. The extraction was blindly validated from a random sample of 67 individual cases, which showed an inter-rater agreement greater than 99% (4121 of 4154 feature matrix instances). We also extracted the following data: type of report (group versus individual), original psychiatric diagnosis before diagnosis of NMDAR-antibody encephalitis, age, sex, presence of neurological features, presence of ovarian teratoma, prior herpes simplex virus encephalitis, and pregnancy. Features from relapses were merged with the first episode data of the respective individual.

Before modelling the primary data formally, we filtered them as means of quality control to better understand the cases with very few lower-level features. We sorted the individual patient data by proxy markers of psychiatric involvement to account for a diversity of sources reporting the patients (reporting in a psychiatry specialist journal, psychiatrist authorship, department of psychiatry authorship). Patients with these markers were placed in the psychiatric-described subgroup and patients without these markers were in the non-psychiatric-described subgroup (appendix).

To develop an operationalised diagnosis classifier, two investigators (AAD and DO) independently compared 14 psychotic and mood disorder diagnoses with the 50 patient-derived lower-level features. They used ICD-10 diagnostic criteria for research throughout; for schizophrenia, the raters also used the DSM-5 classification to compare a unified schizophrenia concept (DSM-5) with subtypes (ICD-10). Because pregnancy and post-partum-associated cases were reported, post-partum psychosis was included as a comparator syndrome. Post-partum psychosis does not have its own category in either diagnostic system,^15^ and therefore a previously reported descriptive cohort was used to identify common lower-level features.^16^ To capture ICD-10 diagnostic criteria for research and DSM-5 inclusion and exclusion criteria (appendix), features were scored as 1 (unequivocally present), 0·5 (possible or transient), 0 (absent) and −2 (would exclude a diagnosis). A consensus categorisation was then finalised.

Next, we used principal component analysis (PCA) in R with the prcomp package and a constrained combination model to compare individual patient data with the operationalised diagnosis classifier. Jaccard indices were generated for the intersection between individual patient data and the 14 operationalised diagnostic categories. These indices evaluated similarities between lower-level features from each case and features expected to be present for each operationalised diagnosis. The Jaccard indices were inputted into the scaled PCA model for dimensionality reduction. PCA transformed these overlaps into a distribution of individual cases and assessed the contribution of each operationalised diagnosis to the chief principal components. The first two principal components (PC1 and PC2) were plotted. Variable loadings for each operationalised diagnostic category were superimposed on this plot.

The constrained combination approach expressed each NMDAR-antibody encephalitis case as a combination of one or more diagnoses, to model whether individuals were best described by one or several diagnoses. For each patient, the features were expressed as the sum of the features of one or more diagnoses. A least-squares approach was used to find a positive scalar for each of the included diagnoses which minimised the error in the patient's feature vector, and the model was penalised if it included features not present or omitted in the operationalised diagnoses. For each patient, we obtained an Akaike information criterion (AIC) index indicating whether their features were best accounted for by one or more diagnoses. AIC comparisons between models generated a ΔAIC.

Finally, we explored inter-relationships with a network analysis. We coded lower-level features as nodes and the co-occurrence frequency between nodes as bidirectional edges. We used a threshold of 10% of the most frequent feature to include more common features. The layout was done with Force Atlas (a force-directed algorithm that minimises edge crossover) in Gephi.^17^

Data were tabulated with Microsoft Excel for Mac (version 16.14.1). Statistical and graphical analyses were done with GraphPad Prism (version 7.0c; GraphPad Software, CA, USA), R (version 3.4.1; R Foundation for Statistical Computing, Vienna, Austria), and MATLAB and Statistics Toolbox Release 2018a (MathWorks, MA, USA). Network analyses were done with Gephi (version 0.9.2 for Mac OS X). An extended version of analytical methods is included in the appendix. The review protocol was prospectively registered with PROSPERO, number CRD42017068981.

### Role of the funding source

The funder of the study had no role in study design, data collection, data analysis, data interpretation, or writing of the report. The corresponding author had full access to all the data in the study. All authors had final responsibility for the decision to submit for publication.

## Results

**Figure 1:**
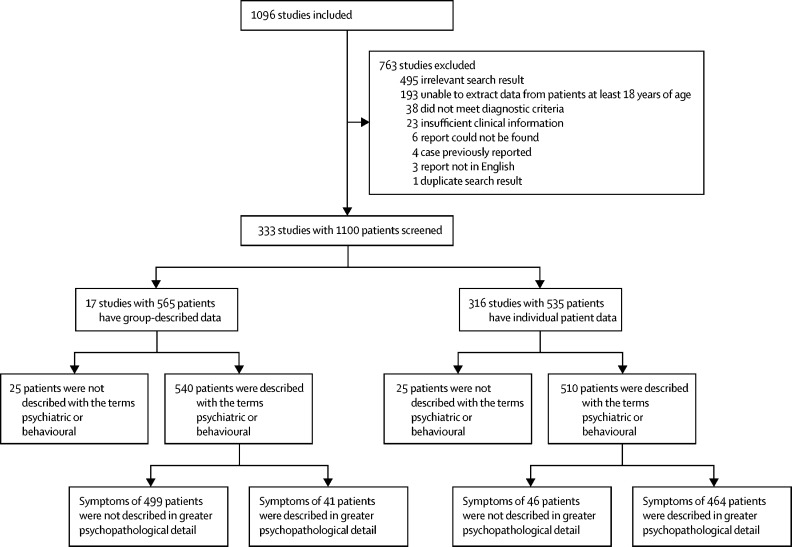
Study selection

Of 1096 records identified in PubMed, 333 satisfied inclusion criteria and described 1100 patients in total (figure 1). Overall, psychiatric symptoms were reported in 1050 (95%) of 1100 patients, including 52 (5%) with isolated psychiatric features. The psychopathology of 505 (46%) patients with reported psychiatric symptoms was described in more detailed terms than psychiatric or behavioural. 464 (91%) of these 505 patients were from papers in which patient data were reported individually. Far greater psychopathological detail was available in the individual patient data reports (both in case reports and series); the remainder of the analyses focused exclusively on these 464 patients, which were highly representative of all group-described data (appendix).

**Figure 2:**
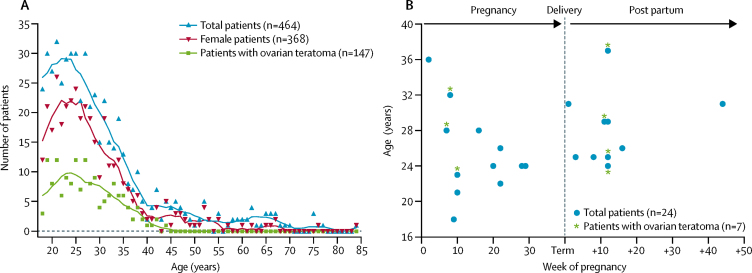
Demographics of 464 patients with N-methyl-D-aspartate receptor-antibody encephalitis (A) Age distribution of patients at onset of N-methyl-D-aspartate receptor (NMDAR)-antibody encephalitis, including female patients and those with ovarian teratomas. Trend lines were smoothed with a second-order function with eight nearest neighbours. (B) Week of pregnancy and ages of patients at onset of pregnancy-associated NMDAR-antibody encephalitis. One case associated with recent miscarriage is not shown.

Overall, the age and sex distribution centred around young women, and the frequency of cases was greatly reduced after 40 years of age (figure 2A). 139 (30%) of 464 cases were associated with ovarian teratoma, seven (2%) with previous herpes simplex virus encephalitis, and 24 (5%) with pregnancy (including 11 [46%] of 24 with post partum; figure 2B). Electroencephalograms (EEGs) were abnormal in 257 (62%) of 412 patients and brain MRI was abnormal in 135 (32%) of 426 patients.

**Figure 3:**
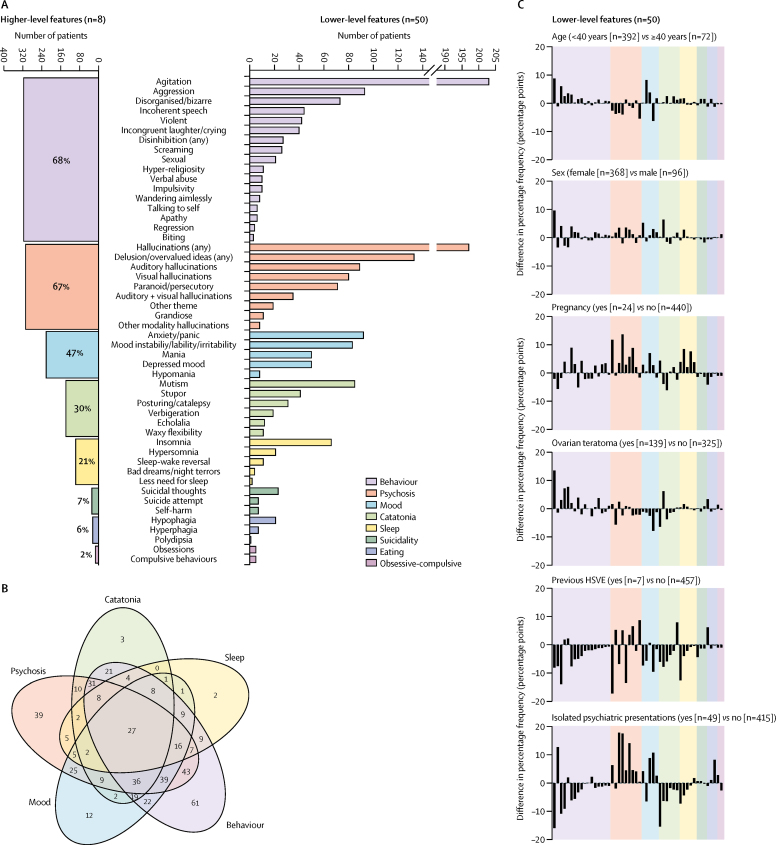
Frequencies and overlaps of higher-level and lower-level psychopathological features from individual patient data 464 patients were from papers in which patient data were reported individually. (A) Number of patients who manifest the eight higher-level and 50 lower-level features. (B) Venn diagram of overlaps between the five most common higher-level features shows frequent coexistence in individual patients. The numbers within the Venn diagram represent numbers of patients who had that pattern of overlaps. (C) Percentage frequencies of the 50 lower-level features in six subgroups of patients classified by age, sex, pregnancy, presence of ovarian teratoma, previous HSVE, and isolated psychiatric presentations. Two-way ANOVA with Bonferroni correction was used to compare the subgroups. None of the subgroups had significantly different frequencies of lower-level features. HSVE=herpes simplex virus encephalitis.

**Figure 4:**
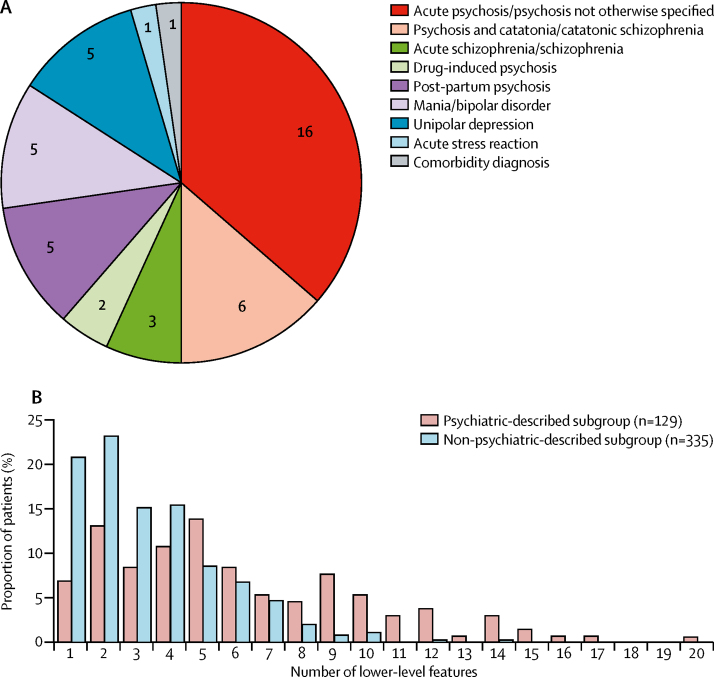
Initial psychiatric diagnoses and lower-level features of patients in the psychiatric-described and non-psychiatric-described subgroups (A) Initial psychiatric diagnoses reported in 44 patients before confirmation of N-methyl-D-aspartate receptor-antibody encephalitis. The comorbidity diagnosis consisted of somatoform disorder NOS, psychotic disorder NOS, and dissociative disorder NOS. (B) Proportion of patients in the psychiatric-described subgroup and non-psychiatric-described subgroup grouped according to the number of psychopathological features used to describe them. NOS=not otherwise specified.

A broad range of psychopathological features was identified, with up to 20 lower-level features per case. The median number of features identified was three (IQR 2–5). The five higher-level categories into which the 464 patients were most frequently grouped (figure 3A) were behaviour (316 [68%]), psychosis (310 [67%]), mood (219 [47%]), catatonia (137 [30%]), and sleep disturbance (97 [21%]). In 334 (74%) of 451 patients, these five features overlapped (figure 3B). The most common overlaps were combinations of mood, psychosis, and behavioural features, often with catatonia. 117 (26%) of 451 patients exhibited only a single higher-level feature. Overall, seven lower-level features explained 77% of the variance in data, defining a salient clinical cluster of common features traversing several higher-level categories: agitation, aggression, hallucinations, delusions, mutism, irritability or mood instability, and depressed mood (appendix). The overall pattern of lower-level features was statistically stable across subgroups classified by age, sex, pregnancy, presence of ovarian teratoma, previous herpes simplex virus encephalitis, and isolated psychiatric presentations (two-way ANOVA p=0·6–0·9, with Bonferroni correction p=1; figure 3C; appendix). This mixed psychopathology was reflected by the initial psychiatric diagnoses reported in 44 patients before confirmation of NMDAR-antibody encephalitis, which included 27 (61%) diagnoses of psychotic disorders (often with catatonia), ten (23%) of classical mood disorders at both ends of polarity with and without psychotic features, and 5 (34%) of post-partum psychosis (figure 4A). More rarely, diagnoses included a drug-induced aetiology.

The polymorphic spectrum of psychiatric diagnoses for which NMDAR-antibody encephalitis was mistaken and the diverse psychopathology observed in many individual patients merited further investigation. After sorting individual patient data by proxy markers of psychiatric involvement, we found that 129 (28%) of 464 patients were in the psychiatric-described subgroup and were enriched for a greater number of lower-level features (figure 4B; appendix). 245 (73%) of the remaining 335 patients in the non-psychiatric-described subgroup were reported using fewer than five lower-level features (figure 4B). The demographics and neurological features of patients in the psychiatric-described subgroup did not differ from those of patients in the non-psychiatric-described subgroup (appendix).

**Figure 5:**
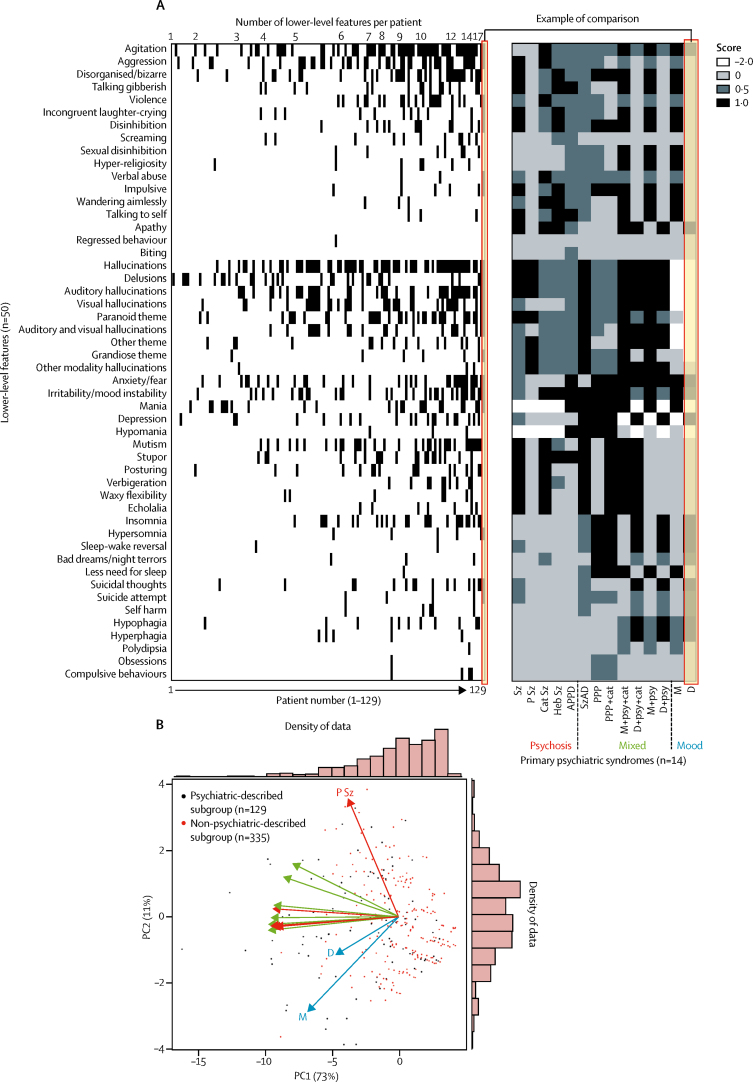
Classification of individual patients using operationalised psychosis and mood spectrum diagnoses (A) Common primary psychiatric syndromes (n=14) were compared with 50 lower-level features to generate a signature for each syndrome. Subsequently, as illustrated by the example of comparison label, each syndrome was compared with each patient in the psychiatric-described subgroup (n=129) and the non-psychiatric-described subgroup (n=335; the heatmap for both subgroups is in the appendix). (B) Principal component analysis of lower-level features. Jaccard indices were generated to assess the overlaps between the 14 operationalised diagnostic categories and individual patient data. Analysis of the variables contributing to the first two principal components (PC1 and PC2) show a clear distribution of patients with N-methyl-D-aspartate receptor-antibody encephalitis defined by a combination of psychosis and mood dimensions. Overlay of the operationalised diagnoses showed orthogonal vectors from mood (blue) and psychotic (red) categories, with mainly mixed disorders (green) within an intermediate space. Histograms on each axis show the density of data mapped across PC1 and PC2. APPD=acute polymorphic psychotic disorder, Cat Sz=catatonic schizophrenia, D=depression, Heb Sz=hebephrenic schizophrenia, M=mania, NOS=not otherwise specified, PC=principal component, PPP=post-partum psychosis, P Sz=paranoid schizophrenia, Sz=schizophrenia, SzAD=schizoaffective disorder, + cat=with catatonia, + psy=with psychotic features.

Next, within both the psychiatric-described and non-psychiatric-described subgroups, each patient was compared with 14 operationalised psychosis and mood spectrum diagnoses (figure 5A), which were chosen to reflect both the prospectively reported diagnoses (figure 4A) and the likely best fits given the observed pattern of lower-level features (figures 3A, 3B). Dimensionality reduction with PCA revealed that PC1 accounted for 73% of variance and PC2 accounted for 11% of variance, together accounting for 84% of the variance in the data. There were no clear, distinct clusters within the PCA plot. Overlay of the operationalised diagnoses showed orthogonal vectors from mood and psychotic categories, with mainly mixed disorders within an intermediate space (figure 5B). This unbiased analysis suggested that overall, patients with NMDAR-antibody encephalitis required combinations of psychotic and mood features to best describe their psychopathology.

**Figure 6:**
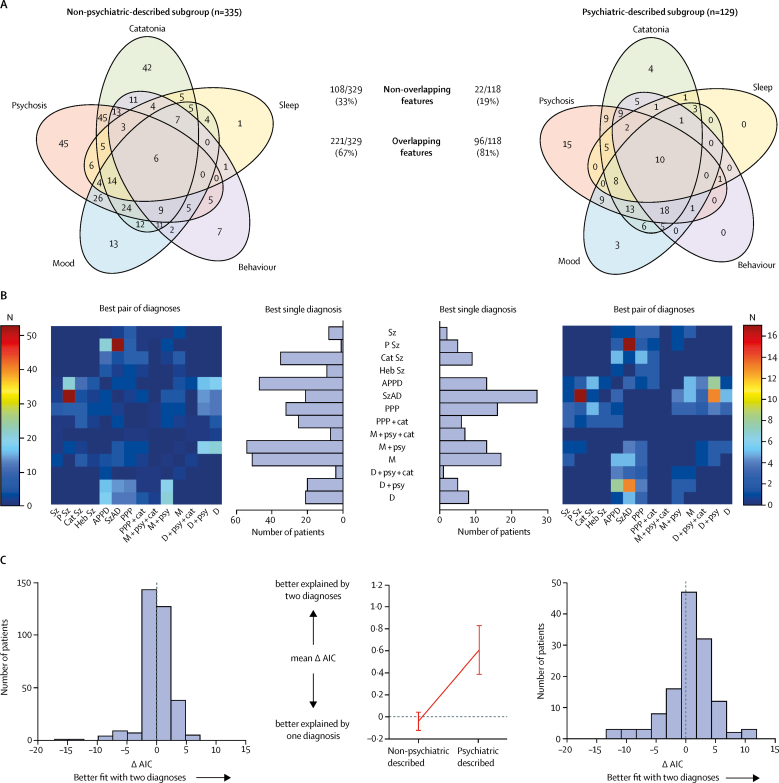

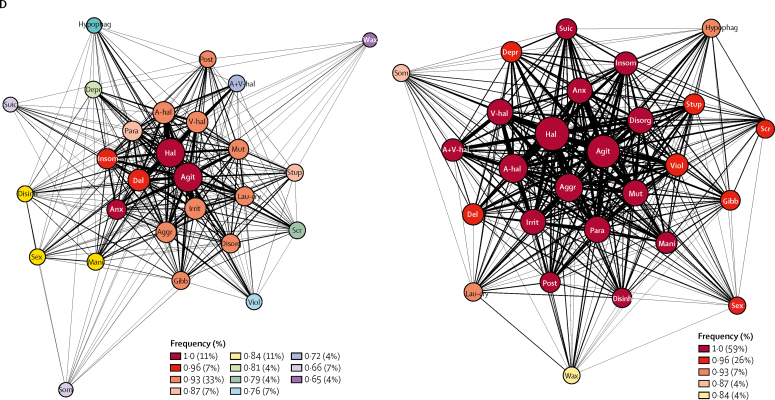
Features of N-methyl-D-aspartate receptor-antibody encephalitis psychopathology in the psychiatric-described and non-psychiatric-described subgroups (A) Venn diagram of overlaps between the five most common higher-level features in psychiatric-described subgroups and non-psychiatric-described subgroups. (B) Estimation of fit using AIC and coherence estimation using network analysis in the non-psychiatricdescribed (n=335) and psychiatric-described (n=129) subgroups. The figure shows the number of patients modelled to each diagnosis (best single diagnosis histogram), and the number of patients with each pair of diagnoses (best pair of diagnoses heatmap). AIC=Akaike information criterion. APPD=acute polymorphic psychotic disorder. Cat Sz=catatonic schizophrenia. D=depression. Heb Sz=hebephrenic schizophrenia. M=mania. PPP=post-partum psychosis. P Sz=paranoid schizophrenia. Sz=schizophrenia. SzAD=schizoaffective disorder. + cat=with catatonia. + psy=with psychotic features. (C) Histograms showing the change in AIC in each group (left and right panels). The mean AIC by two diagnoses than by one. AIC=Akaike information criterion. (D) Network analysis of the 27 lower-level features found at greater than 10% relative frequencies of the most common feature (agitation). The lower-level features are represented as nodes; the size of the nodes is proportionate to the frequency of the feature and the edge thickness is proportionate to the frequency of co-occurrences. The nodes are colour-coded by closeness centrality, a measure of interconnectedness, where one is complete. Agit=agitation. Aggr=aggression. Ahal =auditory hallucinations. A-V hal=auditory and visual hallucinations. Anx=anxiety or fear. Del=delusion. Depr=depression. Disorg=disorganised or bizarre. Disinh=disinhibition. Gibb=talking gibberish. Hal=hallucinations. Insom=insomnia. Irrit=irritability or mood instability. Lau-cry=incongruent laughter-crying. Mani=mania. Mut=mutism. Para=paranoid theme. Phag=hypophagia. Post=posturing. Scr=screaming. Sex=sexual disinhibition. Som=hypersomnia. Stup=stupor. Suic=suicidal thoughts. V-hal=visual hallucinations. Viol=violence. Wax=waxy flexibility.

The overlapping nature of the higher-level features was enriched upon analysis of the psychiatric-described data (221 [67%] of 329 overlaps in non-psychiatric-described subgroup *vs* 96 [81%] of 118 overlaps in psychiatric-described subgroup; χ^2^ test with Yates' continuity correction p=0·0052; figure 6A).

To model the apparent polymorphic appearance of NMDAR-antibody encephalitis psychopathology at an individual patient level, constrained combinations were compared using AIC to test whether two diagnoses would fit each patient better than any single diagnosis alone (figure 6B, 6C). In the non-psychiatric-described subgroup, either one diagnosis or two diagnoses provided equally plausible fits (mean ΔAIC −0·04, SEM 0·073; figure 6C). Consistently, there were several operationalised categories which were best fits including mania (with or without psychotic features), acute polymorphic psychosis, catatonic schizophrenia, and post-partum psychosis (with or without catatonia). However, in the psychiatric-described subgroup, the AIC strongly favoured two diagnoses over one (mean ΔAIC 0·61, SEM 0·153; figure 6C), with 77 (60%) of 129 patients best explained using two diagnoses. Both groups had similar combinations of mood, psychotic, and mixed disorders (figure 6B), and 26 (6%) of 464 patients were better explained by three diagnoses. Constrained combination analysis showed that upon enrichment for more detailed psychopathological descriptions, NMDAR-antibody encephalitis is best characterised as a polymorphic disorder which does not fall exclusively within mood or psychotic disorders, but instead requires complex combinations of both categorisations and often even requires combinations of mixed categorisations.

Finally, network analyses were done to quantify the strength of the connections between multiple lower-level features (figure 6D). Although both the psychiatric-described and non-psychiatric-described subgroups showed network coherence, the psychiatric-described subgroup had a particularly high level of coherence with a very short path length between nodes (path length 1·01 in psychiatric-described subgroup vs path length 1·17 in non-psychiatric-described subgroup) and a markedly high and narrow range of closeness centralities (92% above 0·93 in psychiatric-described subgroup *vs* 51% above 0·93 in the non-psychiatric group).

## Discussion

In a large series of data extracted from 464 individual reports from patients with definite NMDAR-antibody encephalitis and characteristic demographics, the psychopathology was found to be polymorphic and not to respect traditional psychiatric classifications. Rather, it encompassed catatonia, mood, behaviour, and psychosis domains, and was closely represented by a cluster of seven features which crossed the higher-level categorisations: agitation, aggression, hallucinations, delusions, mutism, irritability or mood instability, and depressed mood. This complex transdiagnostic psychopathology was remarkably consistent between patients and stable across diverse subgroups. Overall, the inference of a polymorphic psychopathology that is complex within individuals but fairly consistently reproduced between patients was particularly marked when data were extracted from reports sorted for proxy markers of psychiatric involvement in mental state description, strongly advocating for involvement of psychiatrists in the earliest assessment of patients with NMDAR-antibody encephalitis.^8^, ^10^, ^11^, ^13^, ^14^, ^18^ Our findings should encourage reports to move away from crude, yet frequently reported features such as psychosis and behavioural disturbance. Indeed, patients who are phenotyped with rudimentary features might be more likely to erroneously receive potentially toxic immunotherapies. By contrast, a more nuanced, multifaceted architecture of NMDAR-antibody encephalitis psychopathology could enable psychiatrists to develop a clinical index of suspicion for patients with possible or probable NMDAR-antibody encephalitis, whose care will benefit from CSF analysis, with the aim of expediting diagnosis and thereby enabling earlier immunotherapy administration and improved clinical outcomes.

In addition to the psychopathological features, clinical practice offers other opportunities to differentiate NMDAR-antibody encephalitis from primary psychiatric disorders.^1^, ^4^, ^5^, ^6^, ^9^, ^10^, ^11^, ^12^, ^19^ NMDAR-antibody encephalitis typically emerges over days to weeks, rather than the months and years in most serious mental illnesses.^1^, ^5^ However, temporal information from published literature is scarce, so this is an important area of research for future prospective studies. Given the syndrome of NMDAR-antibody encephalitis has a well-recognised phasic transition between psychiatric features and various neurological features,^4^, ^5^, ^6^ longitudinal observations might show that the psychiatric phase represents a microcosm of this process. This might explain the highly mixed syndromic appearance we observed without incorporating a time component in our analysis, and might more accurately inform the earliest psychopathology of the disease. Other important clues to NMDAR-antibody encephalitis are associated seizures, a movement disorder, and reduction in consciousness.^1^, ^2^, ^3^, ^4^, ^5^, ^6^, ^7^ Nevertheless, as psychiatrists are often the first to see these patients and isolated psychiatric presentations are recognised,^10^, ^11^ a focus on the psychopathology is likely the most effective way to promote prompt recognition of the disorder. Investigations including lumbar puncture and EEG are usually abnormal in NMDAR-antibody encephalitis,^1^, ^2^, ^3^, ^4^, ^5^, ^6^, ^7^ and might provide valuable paraclinical clues. CSF testing has already been safely offered to patients with early-stage, severe mental illnesses.^20^, ^21^

Overall, our observations are supported by a recent systematic review^13^ that listed a wide variety of psychiatric features in patients with NMDAR-antibody encephalitis and helped externally validate our conclusions. However, they did not filter psychiatric features on the basis of quality, and did not analyse the overlaps of psychiatric features between patients or their reproducibility within individual patients. It remains possible that our psychopathological observations show limited specificity for NMDAR-antibody encephalitis, and are consistent with the transdiagnostic nature of features observed both cross-sectionally and longitudinally within several severe mental illnesses.^22^, ^23^ However, outside of NMDAR-antibody encephalitis it is unusual to observe onset of mood, psychotic, and catatonic features over a few weeks, with post-partum psychosis being a key exception. The similar rapid onset and spectrum of psychopathology seen in these two disorders most likely contributed to post-partum onset of NMDAR-antibody encephalitis being mistaken for post-partum psychosis.

With respect to sensitivity, the high coherence of multiple features observed between individual patients, especially within the psychiatrically described cohort, suggests the absence of several higher-level features might have a high negative predictive value for NMDAR-antibody encephalitis. Although 26% of patients exhibited only a single higher-level feature in our analysis, our data (figure 6A) and personal clinical observations suggest this might relate to inexpert psychopathological descriptions.

In addition to the multiple psychopathological features we observed, the distinctive nature of NMDAR-antibody encephalitis is also apparent within the multifaceted, complex accompanying movement disorder,^24^ which typically shows a mixture of dystonia, stereotypies, and chorea. Functional imaging approaches are also beginning to show key characteristic network disruptions.^25^ Perhaps these collective characteristic descriptions represent the underlying mechanism of autoantibody-mediated, NMDAR-specific modulation.^4^, ^26^ Indeed, within early descriptions of NMDAR-antibody encephalitis, striking similarities were noted with other models of NMDAR-specific disruption,^4^ such as experimental and recreational use of phencyclidine and ketamine.^27^ This hypothesised target-specific psychopathology might therefore not translate to other autoantibody-mediated phenotypes. Yet the identified clinical overlaps—most obviously with post-partum psychosis—might implicate reversible NMDAR-dominant receptor-mediated network disruptions in these more common conditions secondary to other non-immune mechanisms.^15^, ^28^ Overall, the potential direct link between a molecule and a clinically defined mental state presents an exciting and rare opportunity to relate a precise molecular mechanism to psychopathology, a long-term goal in psychiatric research.^23^

Our study design has several potential limitations. Firstly, sole inclusion of adults means that results cannot be generalised to the paediatric population. This is clinically consistent with the traditional division of health services at 18 years of age. Also, as the movement disorder is more prominent in children with NMDAR-antibody encephalitis onset, their outcomes might benefit less markedly from accurate psychopathology recognition.^5^ Secondly, the under-reporting of lower-level features in many of the included reports might reduce our ability to generate a consistent complex psychopathology in NMDAR-antibody encephalitis. Indeed, enrichment for psychiatric-described cases, which reported higher numbers of lower-level features, generated greater coherence between features. Case reports might predispose towards outlying cases, so they remain as a possible confounder of our approach, but deviations in demographics, aetiology, or neurological profiles were absent (appendix), highlighting no evidence of a systematic reporting bias. Thirdly, our study focused on reports of NMDAR-antibody encephalitis without identical psychopathological analyses in several other disease cohorts. This limits our ability to conclude definitively that NMDAR-antibody encephalitis can be differentiated accurately from other psychiatric conditions on the basis of psychopathological features alone. To address this limitation, the field would benefit from a comparable cross-sectional cohort study of primary psychiatric disorders; however, this study might take many centres and years to generate sufficiently large cohorts of patients with NMDAR-antibody encephalitis. Systematic electronic care records will facilitate the study of clinical populations in the future.

Overall, NMDAR-antibody encephalitis encompasses multiple psychopathological domains which are traditionally considered mutually exclusive. Combinations of psychotic, mood, and mixed disorders are required to adequately describe the disease, consistent with real world clinical experience. By combining the characteristic epidemiology with the core clinical skill of descriptive psychopathology, psychiatrists can generate an appropriate index of suspicion for the likelihood of NMDAR-antibody encephalitis, without reliance on frank neurological features. If validated and systematically applied, this approach might provide adequate diagnostic sensitivity to hasten clinical discussions about further investigations, most importantly paired serum and CSF testing and the appropriate setting of care and timing of immunotherapies.

## References

[1] Dalmau J, Tüzün E, Wu HY (2007). Paraneoplastic anti-N-methyl-D-aspartate receptor encephalitis associated with ovarian teratoma. Ann Neurol.

[2] Armangue T, Leypoldt F, Málaga I (2014). Herpes simplex virus encephalitis is a trigger of brain autoimmunity. Ann Neurol.

[3] Hacohen Y, Deiva K, Pettingill P (2013). N-methyl-D-aspartate receptor antibodies in post-herpes simplex virus encephalitis neurological relapse. Mov Disord.

[4] Dalmau J, Lancaster E, Martinez-Hernandez E, Rosenfeld MR, Balice-Gordon R (2011). Clinical experience and laboratory investigations in patients with anti-NMDAR encephalitis. Lancet Neurol.

[5] Titulaer MJ, McCracken L, Gabilondo I (2013). Treatment and prognostic factors for long-term outcome in patients with anti-NMDA receptor encephalitis: an observational cohort study. Lancet Neurol.

[6] Irani SR, Bera K, Waters P (2010). N-methyl-D-aspartate antibody encephalitis: temporal progression of clinical and paraclinical observations in a predominantly non-paraneoplastic disorder of both sexes. Brain.

[7] Lejuste F, Thomas L, Picard G (2016). Neuroleptic intolerance in patients with anti-NMDAR encephalitis. Neurol Neuroimmunol Neuroinflamm.

[8] Moran N, Munro N, Lawson K, David AS (2014). Safe management of psychiatrically disturbed patients on non-psychiatric wards in the UK. BMJ.

[9] Graus F, Titulaer MJ, Balu R (2016). A clinical approach to diagnosis of autoimmune encephalitis. Lancet Neurol.

[10] Kayser MS, Titulaer MJ, Gresa-Arribas N, Dalmau J (2013). Frequency and characteristics of isolated psychiatric episodes in anti-N-methyl-D-aspartate receptor encephalitis. JAMA Neurol.

[11] Steiner J, Walter M, Glanz W (2013). Increased prevalence of diverse N-methyl-D-aspartate glutamate receptor antibodies in patients with an initial diagnosis of schizophrenia: specific relevance of IgG NR1a antibodies for distinction from N-methyl-D-aspartate glutamate receptor encephalitis. JAMA Psychiatry.

[12] Herken J, Prüss H (2017). Red flags: clinical signs for identifying autoimmune encephalitis in psychiatric patients. Front Psychiatry.

[13] Warren N, Siskind D, O'Gorman C (2018). Refining the psychiatric syndrome of anti-N-methyl-D-aspartate receptor encephalitis. Acta Psychiatr Scand.

[14] Scott JG, Gillis D, Ryan AE (2018). The prevalence and treatment outcomes of antineuronal antibody-positive patients admitted with first episode of psychosis. BJPsych Open.

[15] Jones I, Chandra PS, Dazzan P, Howard LM (2014). Bipolar disorder, affective psychosis, and schizophrenia in pregnancy and the pot-partum period. Lancet.

[16] Heron J, McGuinness M, Blackmore ER, Craddock N, Jones I (2008). Early postpartum symptoms in puerperal psychosis. BJOG.

[17] Bastian M, Heymann S, Jacomy M (2009). Gephi: an open source software for exploring and manipulating networks. Proc Int AAAI Conf Weblogs Soc Media.

[18] Lennox BR, Palmer-Cooper EC, Pollak T (2017). Prevalence and clinical characteristics of serum neuronal cell surface antibodies in first-episode psychosis: a case-control study. Lancet Psychiatry.

[19] Irani SR, Gelfand JM, Al-Diwani A, Vincent A (2014). Cell-surface central nervous system autoantibodies: clinical relevance and emerging paradigms. Ann Neurol.

[20] Endres D, Perlov E, Baumgartner A (2015). Immunological findings in psychotic syndromes: a tertiary care hospital's CSF sample of 180 patients. Front Hum Neurosci.

[21] Oviedo-Salcedo T, de Witte L, Kümpfel T (2018). Absence of cerebrospinal fluid antineuronal antibodies in schizophrenia spectrum disorders. Br J Psychiatry.

[22] Caspi A, Moffitt TE (2018). All for one and one for all: mental disorders in one dimension. Am J Psychiatry.

[23] Stanghellini G, Broome MR (2018). Psychopathology as the basic science of psychiatry. Br J Psychiatry.

[24] Varley JA, Webb A, Balint B (2018). The movement disorder associated with NMDAR antibody-encephalitis is complex and characteristic: an expert video-rating study. J Neurol Neurosurg Psychiatry.

[25] Peer M, Prüss H, Ben-Dayan I, Paul F, Arzy S, Finke C (2017). Functional connectivity of large-scale brain networks in patients with anti-NMDA receptor encephalitis: an observational study. Lancet Psychiatry.

[26] Jézéquel J, Johansson EM, Leboyer M, Groc L (2018). Pathogenicity of antibodies against NMDA receptor: molecular insights into autoimmune psychosis. Trends Neurosci.

[27] Vinckier F, Gaillard R, Palminteri S (2015). Confidence and psychosis: a neuro-computational account of contingency learning disruption by NMDA blockade. Mol Psychiatry.

[28] Bergink V, Armangue T, Titulaer MJ, Markx S, Dalmau J, Kushner SA (2015). Autoimmune encephalitis in postpartum psychosis. Am J Psych.

