# Supplementary material for: The psychopathology of NMDAR-antibody encephalitis in adults: a systematic review and phenotypic analysis of individual patient data
Source: Lancet Psychiatry. 2019 Mar;6(3):235–46. doi: 10.1016/S2215-0366(19)30001-X (PMC6384244; doi:10.1016/S2215-0366(19)30001-X)

THE LANCET
Psychiatry

Supplementary appendix

This appendix formed part of the original submission and has been peer reviewed. We post it as supplied by the authors.

Supplement to: Al-Diwani A, Handel A, Townsend L, et al. The psychopathology of anti-NMDA receptor encephalitis in adults: a systematic review and phenotypic analysis of individual patient data. *Lancet Psychiatry* 2019; published online Feb 11. http://dx.doi.org/10.1016/S2215-0366(19)30001-X.

Supplementary methods

Cumulative proportion of cases accounted for by lower level features

The proportion of cases accounted for by increasing number of lower level features was calculated by ranking lower level features by their frequency in the cohort. This was then used to identify the cumulative proportion of cases that were positive for at least one of those features while systematically increasing the number of lower level features included. This is shown in Supplementary Figure 1A.

2-way ANOVA on summary data

A 2-way ANOVA was performed in R using the aov function to fit subgroup labels and lower level features. The P-value for the interaction term was reported in each case and adjusted where appropriate for multiple hypothesis testing using Bonferroni and Benjamini-Hochberg correction.

Principal component analysis (PCA)

The prcomp function in R was used for dimensionality reduction on individual case (C) fits to comparator diagnoses (D). The overlap with comparator diagnoses was assessed in each case for each diagnosis using the Jaccard Index.

$$J(C,D) = \frac{|C \cap D|}{|C \cup D|}$$

This generated a matrix of case-by-diagnosis Jaccard indices. Prcomp was used for dimensionality reduction on this matrix using the option to pre-scale data. Individual cases were projected into the first and second principal components on a scatter plot. The weightings of individual comparator diagnoses for the first and second principal components were assessed from the PCA model rotations and plotted as vectors.

Density plots of individual cases across the first and second principal components were generated by the R function hist.

Are psychiatric features better explained by single or multiple operationalised diagnoses?

In this analysis, we asked whether each person's combination of psychiatric features were better explained by a single operationalised diagnosis, or by a combination of two or three psychiatric diagnoses. To find the best single-diagnosis, the patient's feature vector was regressed against each of the operationalised diagnosis vectors. Least squares regression estimates a single coefficient for each possible diagnosis, and a measure of the error for that diagnosis, i.e. how poorly that diagnosis fits the features. The diagnosis with the smallest error was chosen. To find the best double-diagnosis, the feature vector was regressed against each pair of possible operationalised diagnoses. Here, regression estimates two coefficients, one for the contribution of each of the two diagnoses, and an error. The pair with the smallest error was chosen. A similar analysis was performed for combinations of three operationalised diagnoses.

To determine whether the patient was better fit by one or by two diagnoses, we penalised the fitting error for each of these two models by the number of free parameters. This is because fits will always be better when a combination of two diagnoses are permitted, simply because of the extra degree of freedom in fitting. The Akaike information criterion (AIC) offers a formal way to judge whether adding the extra diagnosis is 'worth it'. A more negative value of AIC indicates that a model is better than another. We used the formula:

$$AIC = 2k + N \log \frac{2\pi SSE}{N}$$

where k is the number of free parameters (1 or 2, for the 1-diagnosis or 2-diagnosis models respectively) and N is the number of features being fitted (i.e. 50). To compute the evidence (log likelihood ratio) in favour of using two diagnoses, the AIC for the 2-diagnosis was subtracted from the AIC for the 1-diagnosis model, and this difference was added up over all patients in the group. A more negative value corresponds to the 2-diagnosis model being more likely.

Network analysis of lower level features

A correlation matrix of the 50 lower level features was generated for each of the psychiatric- and non-psychiatric-described sub-groups. This was used to define undirected edge weights for each source and target node combination which were then imported into Gephi. Features that were >10% of the rate of the most

common feature were included in the analysis, the remainder were removed. Each sub-group network was visualised using the Force Atlas algorithm with the following settings: inertia 0.1, repulsion strength 700000, attraction strength 8.0, maximum displacement 10.0, auto stabilize function on, autostab strength 500, autostab sensibility 0.2, gravity 25.0, attraction distrib off, adjust by sizes on and speed 1. The following analyses were run using inbuilt packages: average degree, average weighted degree, graph density, modularity (threshold iterations of 0.7, 0.8 and 0.9), average clustering coefficient and average path length. The network diameter package was used to calculate closeness and betweenness centrality distributions from which mean, median and range values were derived.

Supplementary material

Supplementary table 1. Operationalised diagnosis matrix. Diagnoses are listed in rows ordered from pure psychotic (top) to pure mood (bottom) with mixed in between. 50 lower level features of NMDAR-Ab-E are in columns ordered within higher level categories in decreasing frequency. Cells are coloured as per respective guidelines: white for -2 (exclude diagnosis), light grey for 0 (absent), dark grey for 0.5 (possible and/or transient) and black for 1 (present). Extracted from figure 5A and rotated for clarity.

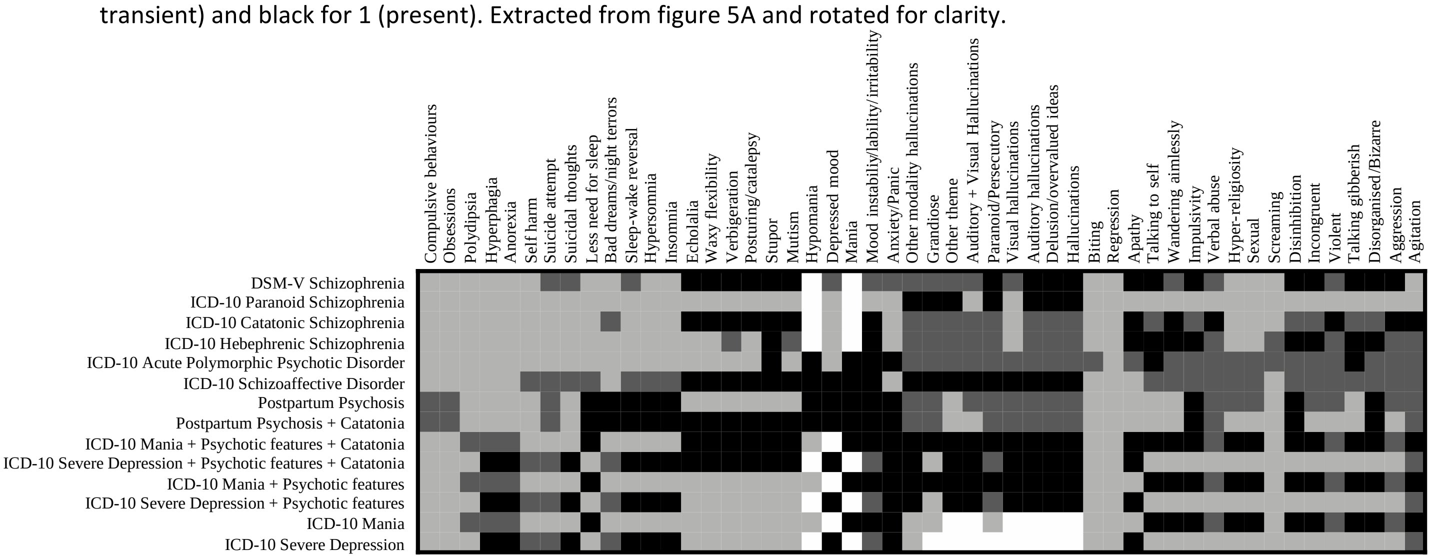

Supplementary table 2. Study characteristics of included studies. *age-specific data extracted from figure.

Author et al. (year)	PMID	Study type	Number of participants (≥ 18 years)	Number of females	Number of males	Age (Individual ages or group mean)
**Group data**						
Graus F et al. (2008)	18794496	Case series	4	3	1	39·5
Pham HP et al. (2011)	21898576	Case series	7	7	0	23·6
Schmitt SE et al. (2012)	22933737	Case series	23	19	4	24
Titulaer MJ et al. (2013)*	23290630	Observational cohort study	364	186	55	28·4
Titulaer MJ et al. (2013)	23946310	Observational cohort study	31	17	14	52
Tabata E et al. (2014)	24296881	Case series	3	3	0	25·3
Köhler W et al. (2014)	24589025	Case series/ Retrospective pilot study	9	8	1	32
Lim JA et al. (2014)	24829602	Case series	32	15	17	41·5
Wegner F et al. (2014)	24950993	Case control study	5	5	0	36·5
Sarkis RA et al. (2014)	25171259	Retrospective chart review	5	3	2	43·2
Gong YH et al. (2015)	26521801	Case series	5	5	0	27·6
Liba Z et al. (2016)	26941012	Case control study	4	4	0	20·5
Novy J et al. (2016)	27397089	Case series	4	4	0	24.5
McKeon GL et al. (2016)	27546201	Case series	4	3	1	24
Huang Q et al. (2016)	27632180	Case series	14	8	6	28·3
Deng B et al. (2017)	28284352	Case control study	40	20	20	28
Wang BJ et al. (2017)	28477682	Case series	9	6	3	35·2
**Individual data**						

Dalmau J et al. (2007)	17262855	Case series	10	10	0	30·3
Sansing LH et al. (2007)	17479076	Case report	1	1	0	34
Iizuka T et al. (2008)	17898324	Case series	3	3	0	28·7
Seki M et al. (2008)	18032452	Case report	1	1	0	18
Novillo-López ME et al.	18299525	Case report	1	1	0	53
Kleinig TJ et al. (2008)	18442127	Case report	3	3	0	26·3
Wilder-Smith EP et al. (2008)	18657715	Case report	1	0	1	59
Nasky KM et al. (2008)	18704025	Case report	1	1	0	Not extractable
Eker A et al. (2008)	18708569	Case report	1	0	1	30
Shimazaki H et al. (2008)	18779433	Case report	1	1	0	30
Shindo A et al. (2009)	19033688	Case series	2	2	0	42
Ishiura H et al. (2008)	19047564	Case report	1	1	0	30·5
Khadem GM et al. (2009)	19290983	Case report	1	1	0	57
Niehusmann P et al. (2009)	19364930	Prospective cohort study	4	4	0	27
Kort DH et al. (2009)	19623000	Case report	1	1	0	20
Tang T et al. (2009)	19639467	Case report	1	1	0	24
Labate A et al. (2009)	19713171	Case report	1	1	0	26
Gable MS et al. (2009)	19718525	Case series	6	5	1	24
Bayreuther C et al. (2009)	19736168	Case report	1	1	0	25
Parratt KL et al. (2009)	19740054	Case report	1	1	0	21
Davies G et al. (2010)	20016378	Observational cohort study	6	4	2	27·5
Kurian M et al. (2010)	20065141	Case report	1	1	0	23
Ferioli S et al. (2010)	20142537	Case report	1	1	0	26
Naeije G et al. (2010)	20347215	Case report	1	1	0	29
Fawcett RG et al. (2010)	20409449	Case report	1	1	0	28
Gold D et al. (2010)	20479353	Case report	1	0	1	18
Mohr BC et al. (2010)	20479604	Case series	1	1	0	23

Irani SR et al. (2010)	20511282	Case series	34	23	11	28·5
Tan A et al. (2010)	20605463	Case series	2	2	0	27
Kumar MA et al. (2010)	20625099	Case series	1	1	0	21
Maeder-Ingvar M et al. (2011)	20667855	Case report	1	1	0	25
Lo JW et al. (2010)	20683078	Case report	1	1	0	27
Varvat J et al. (2010)	20715742	Case report	1	1	0	41
Camdessanché JP et al. (2011)	20722705	Case report	1	1	0	25
Kleyensteuber B et al. (2010)	20731268	Case report	1	1	0	47
Braakman HM et al. (2010)	20819992	Case report	1	0	1	35
Johnson N et al. (2010)	20826712	Case report	1	1	0	21
Tachibana N et al. (2010)	20930449	Case report	1	1	0	23·7
Prüss H et al. (2010)	21060097	Retrospective chart analysis	6	6	0	18
Kirkpatrick MP et al. (2011)	21190901	Case report	1	1	0	19
Asai S et al. (2011)	21195970	Case series	2	2	0	26·5
Hara M et al. (2011)	21290144	Case report	1	1	0	65
Kung DH et al. (2011)	21300200	Case report	1	0	1	24
Day GS et al. (2011)	21318640	Case series	3	2	1	47·7
Frechette ES et al. (2011)	21321356	Case report	1	0	1	18
Tojo K et al. (2011)	21327179	Case report	1	0	1	19
Alexopoulos H et al. (2011)	21384161	Case report	1	1	0	42
Xia C et al. (2011)	21502593	Case report	1	1	0	27
Caballero PE et al. (2011)	21625778	Case report	1	1	0	28
Chia PL et al. (2013)	21671956	Case series	2	2	0	26·5
Naoura I et al. (2011)	21704961	Case report	1	1	0	27
Sacré K et al. (2011)	21757319	Case series	2	2	0	27
Smith JH et al. (2011)	21825245	Case report	1	1	0	27
Pryzbylkowski PG et al. (2011)	21918158	Case series	2	1	1	21
Finke C et al. (2012)	21933952	Case series	9	8	1	28·4

Pascual-Ramírez J et al. (2011)	21945049	Case series	2	2	0	30
Torgovnick J et al. (2011)	21948933	Case report	1	0	1	52
Barry H et al. (2011)	21984802	Case series	4	4	0	24
Lee M et al. (2011)	21992741	Case report	1	1	0	41
Uruha A et al. (2012)	22047651	Case report	1	0	1	68
Yu AY et al. (2011)	22054628	Case report	1	1	0	29
Yen L et al. (2011)	22132359	Case report	1	0	1	22
Lekoubou A et al. (2012)	22182357	Case report	1	1	0	34
Mesquita J et al. (2011)	22231330	Case report	1	1	0	21
Dou YH et al. (2012)	22286659	Case report	1	1	0	29
Pinho J et al. (2012)	22353328	Case series	2	1	1	26
Rao RM et al. (2011)	22361499	Case report	1	1	0	42
Tanyi JL et al. (2012)	22390222	Case series	3	3	0	28
Salazar R et al. (2012)	22390888	Case report	1	1	0	37
Evoli A et al. (2012)	22392580	Case report	1	1	0	29
Batra R et al. (2012)	22411418	Case report	1	1	0	27
Matsumoto T et al. (2012)	22443247	Case report	1	0	1	34
Dean Z et al. (2012)	22450464	Case report	1	1	0	18
Pennington C et al. (2012)	22459355	Case report	1	1	0	23
Leypoldt F et al. (2012)	22566598	Case series	6	4	0	31
Tsutsui K et al. (2012)	22569157	Case series	4	3	1	24·5
Perogamvros L et al. (2012)	22596107	Case report	1	1	0	23·8
Wali SM et al. (2011)	22699468	Case report	1	1	0	22
Shaaban HS et al. (2012)	22705616	Case report	1	1	0	25
Ikeguchi R et al. (2012)	22728495	Case report	1	1	0	19
McCarthy A et al. (2012)	22752087	Case report	1	1	0	32
Tarula E et al. (2012)	22764365	Case report	1	0	1	31
Dulcey I et al. (2012)	22770971	Case report	1	1	0	20
See AT et al. (2012)	22779979	Case report	1	1	0	31

Dabner M et al. (2012)	22833082	Case series	3	3	0	27·7
Suzuki H et al. (2013)	22851287	Case report	1	1	0	50
Roberts R et al. (2012)	22859813	Case report	1	1	0	33
Reid DK et al. (2013)	22952330	Case report	1	1	0	19
Aoki H et al. (2012)	23032251	Case report	1	1	0	21
Holzer FJ et al. (2012)	23051892	Case series	6	5	1	32·8
Kataoka H et al. (2012)	23112259	Case series	1	1	0	46
Sakamoto H et al. (2013)	23128856	Case series	4	0	4	32.3
Hinson HE et al. (2013)	23229019	Case report	1	1	0	31
Hansen HC et al. (2013)	23318518	Case report	1	1	0	25
Sanmaneechai O et al. (2013)	23419475	Case report	1	1	0	19
Young PJ et al. (2013)	23432495	Case series	5	5	0	26
Ryan SA et al. (2013)	23507817	Case report	1	1	0	37
Ramanathan S et al. (2013)	23528411	Case series	1	1	0	31
Gumbinger C et al. (2013)	23635958	Case report	1	1	0	38
Sorita A et al. (2013)	23648916	Case report	1	1	0	35
Erdoğan C et al. (2013)	23670406	Case report	1	0	1	42
Kumar R et al. (2013)	23720469	Case report	1	1	0	26
Dericioglu N et al. (2013)	23773862	Case series	2	2	0	25·5
Kayser MS et al. (2013)	23877059	Observational cohort study	15	13	2	29·8
Punja M et al. (2013)	23962100	Case series	2	1	1	24
Verfaillie L et al. (2013)	23967726	Case report	1	1	0	18
Hopkins SA et al. (2013)	23997078	Case report	1	1	0	34
Tidswell J et al. (2013)	24108083	Case report	1	1	0	27
Maraka S et al. (2013)	24195175	Case report	1	1	0	33
Cassa RS et al. (2013)	24201920	Case report	1	0	1	31
Marques IB et al. (2014)	24210076	Case report	1	0	1	30
Di Capua D et al. (2013)	24317133	Case report	1	0	1	66

Armangue T et al. (2014)	24318406	Case report	1	0	1	24
Ishikawa Y et al. (2013)	24334591	Case report	1	1	0	26
Thomas L et al. (2014)	24355654	Retrospective data analysis	1	0	1	77
Beatty CW et al. (2014)	24381709	Case report	1	1	0	45
Seifi A et al. (2013)	24392258	Case report	1	1	0	19
Lapébie FX et al. (2014)	24433363	Case report	1	1	0	24
Viaccoz A et al. (2014)	24443452	Case series	13	0	13	33·6
Finke C et al. (2014)	24619988	Case report	1	0	1	67
Thilagavathi TV et al. (2013)	24632875	Case report	1	0	1	24
Gulyayeva NA et al. (2014)	24635944	Case series	2	2	0	20·5
Titulaer MJ et al. (2014)	24700511	Case series	14	10	0	36·8
Jagota P et al. (2014)	24706012	Case report	1	1	0	18
Mariotto S et al. (2014)	24707266	Case report	1	1	0	32
Kuppuswamy PS et al. (2014)	24731834	Case series	2	1	1	32·5
Lin JJ et al. (2014)	24739379	Comparative case series	5	4	0	23
Cleverly K et al. (2014)	24773063	Case series	2	2	0	23·5
Day GS et al. (2014)	24781184	Case control study	5	5	0	28·2
Howard CM et al. (2014)	24798839	Case series	5	3	0	28
Çoban A et al. (2014)	24825964	Retrospective case-control analysis	1	0	1	58
MacMahon M et al. (2013)	24829820	Case report	1	1	0	21
Yuan N et al. (2013)	24922990	Case report	1	1	0	22
Yilmaz B et al. (2014)	25033868	Case report	1	1	0	23
Sethi NK et al. (2014)	25082492	Case report	1	1	0	30
Kattepur AK et al. (2014)	25106449	Case report	1	1	0	36
Chen B et al. (2014)	25187817	Case report	1	1	0	38

Colley S et al. (2014)	25193812	Case report	1	1	0	30
Nolan B et al. (2014)	25227654	Case report	1	0	1	20
Koksal A et al. (2015)	25267686	Case report	1	1	0	25
Bach LJ et al. (2014)	25323083	Case series	3	3	0	25
Jørgensen A et al. (2015)	25338667	Case series	1	0	1	27
Prüss H et al. (2014)	25340067	Case report	1	0	1	75
Taraschenko O et al. (2014)	25340077	Case report	1	0	1	65
Li S et al. (2015)	25377541	Case report	1	1	0	23
Schäbitz WR et al. (2014)	25378669	Case report	1	1	0	76
Fleischmann R et al. (2015)	25384024	Case report	1	1	0	33
Keller S et al. (2014)	25400967	Case report	1	1	0	32
Azizyan A et al. (2014)	25426239	Case report	1	1	0	19
Freeman JW et al. (2014)	25490799	Case report	1	0	1	37
Tobin WO et al. (2014)	25566417	Case report	1	0	1	21
Yoshimura B et al. (2015)	25620565	Case series	2	2	0	47·5
Lee EM et al. (2014)	25625091	Case series	2	2	0	26
Bradley L et al. (2014)	25626115	Case report	1	1	0	52
VanHaerents S et al. (2014)	25667873	Case report	1	1	0	27
Power L et al. (2014)	25668045	Case report	1	1	0	26
Lamale-Smith LM et al. (2015)	25710615	Case report	1	1	0	24
Jensen P et al. (2015)	25713112	Case report	1	0	1	35
Kadoya M et al. (2015)	25743014	Case report	1	1	0	48
Chan LW et al. (2015)	25820508	Case report	1	1	0	23
Ioannidis P et al. (2015)	25837318	Case report	1	1	0	29
Monteiro VL et al. (2015)	25860567	Case report	1	0	1	20
Ziaeian B et al. (2015)	25873835	Case report	1	1	0	19
Takeda A et al. (2014)	25876479	Case report	1	0	1	35
Orengo JP et al. (2015)	25884011	Case report	1	1	0	29
Pistacchi M et al. (2015)	25894351	Case report	1	0	1	30

Lu J et al. (2015)	25903201	Case report	1	1	0	36
Gahr M et al. (2015)	25917068	Case report	1	0	1	34
Wang J et al. (2015)	25921549	Case series	2	1	1	38·5
Mangalwedhe SB et al. (2015)	25923864	Case report	1	1	0	35
Lalanne L et al. (2015)	25923872	Case report	1	1	0	31
Shahani L et al. (2015)	25926583	Case report	1	1	0	26
Shaikh MA et al. (2015)	25937654	Case report	1	1	0	32
Reilly-Shapiro C et al. (2016)	25962778	Case report	1	1	0	18
Vahter L et al. (2015)	25988034	Case report	1	1	0	29
Amer S et al. (2015)	26019434	Case report	1	1	0	21
Kurita D et al. (2015)	26037482	Case report	1	1	0	23
Jones SV et al. (2014)	26037868	Case report	1	1	0	39
Huang C et al. (2015)	26089673	Case report	1	1	0	25
Mathis S et al. (2015)	26131809	Case report	1	1	0	21
Imai K et al. (2015)	26136883	Case report	1	1	0	39
Morris NA et al. (2016)	26139017	Case report	1	1	0	67
Wang RJ et al. (2015)	26152327	Case report	1	1	0	24
Hur J et al. (2015)	26157594	Case report	1	1	0	36
Kim H et al. (2015)	26157669	Case report	1	0	1	31
Kim J et al. (2015)	26157673	Case report	1	1	0	28
Bergink V et al. (2015)	26183699	Case series	2	2	0	28
Kiani R et al. (2015)	26191422	Case series	2	1	1	37
Heekin RD et al. (2015)	26199781	Case report	1	1	0	24
Brozzi MK et al. (2015)	26222396	Case report	1	1	0	25
Endres D et al. (2015)	26231521	Case report	1	1	0	31
Kadoya M et al. (2015)	26234239	Case report	1	0	1	46
Liu J et al. (2015)	26277996	Case series	8	4	4	27·7
Malayev Y et al. (2015)	26322937	Case report	1	1	0	33
van Mierlo (2015)	26371800	Case series	3	2	1	26·3

Kamble N et al. (2015)	26448226	Case series	4	4	0	21
Liu H et al. (2015)	26475263	Case series	2	2	0	26·5
Çoban A et al. (2016)	26481863	Case series	2	0	2	63
Armangue T et al. (2015)	26491084	Case series	2	2	0	51·5
Sühs KW et al. (2015)	26622479	Case series	3	3	0	33·7
Zhou SX et al. (2015)	26823917	Case report	1	1	0	31
Wong D et al. (2014)	26839775	Case report	1	1	0	21
Chanson et al. (2016)	26922283	Case report	1	1	0	28
Feigal J et al. (2016)	26922652	Case report	1	1	0	23
Sunwoo JS et al. (2015)	26959858	Case report	1	1	0	27
Rajahram GS et al. (2015)	26988212	Case report	1	1	0	35
Loughan AR et al. (2016)	26998574	Case report	1	0	1	42
Jeraiby M et al. (2016)	27019996	Case report	1	1	0	62
Vargas RJ et al. (2016)	27022186	Case report	1	1	0	25
Mythri SV et al. (2016)	27114630	Case report	1	1	0	27
Raynor G et al. (2016)	27148913	Case report	1	1	0	24
Clara JA et al. (2016)	27154777	Case report	1	1	0	31
Mehr SR et al. (2016)	27190663	Case report	1	1	0	31
Williams TJ et al. (2016)	27271951	Case report	1	1	0	55
Afanasiev V et al. (2016)	27281530	Case report	1	1	0	51
Hinkle CD et al. (2017)	27291044	Case series	1	1	0	18
Weaver M et al. (2016)	27301666	Case report	1	1	0	35
Lasoff DR et al. (2016)	27330659	Case report	1	1	0	23
Seifi A et al. (2016)	27343709	Case report	1	1	0	23
Chawla R et al. (2016)	27393847	Case report	1	1	0	29
Jones BP et al. (2017)	27425649	Case report	1	1	0	29
Parfene C et al. (2016)	27444052	Case report	1	1	0	34
Luo JJ et al. (2016)	27456878	Case report	1	1	0	19
Maccaferri GE et al. (2016)	27468380	Case report	1	1	0	22

Yoga B et al. (2014)	27489663	Case report	1	1	0	21
Hegen H et al. (2016)	27502387	Case report	1	1	0	31
Tantipalakorn C et al. (2016)	27511754	Case report	1	1	0	23
Solís N et al. (2016)	27529308	Case report	1	1	0	29
Behrendt V et al. (2016)	27533122	Case series	2	2	0	25
Halbert RK et al. (2016)	27579962	Case report	1	1	0	33
Lejuste F et al. (2016)	27606355	Case series	21	18	3	27
Baker J et al. (2016)	27625730	Case series	2	2	0	27·5
Milovac Ž et al. (2016)	27658841	Case report	1	1	0	34
Mimbella PC et al. (2016)	27673017	Case report	1	1	0	25
Kremm LA et al. (2016)	27673080	Case report	1	1	0	26
Medepalli K et al. (2016)	27708781	Case report	1	1	0	23
McIvor K et al. (2017)	27762163	Case report	1	0	1	19
Abdul-Rahman ZM et al. (2016)	27776544	Case report	1	1	0	25
Peng Y et al. (2017)	27778112	Case report	1	0	1	51
Shi Y et al. (2017)	27821481	Case report	1	1	0	20
Schein F et al. (2017)	27826871	Case report	1	1	0	52
Zhang W et al. (2017)	27899309	Case report	1	1	0	18
Iriondo O et al. (2017)	27919412	Case report	1	0	1	19
Singh G et al. (2016)	27922228	Case report	1	1	0	23
Pattanayak P et al. (2017)	27941376	Case report	1	1	0	33
Mathai SK et al. (2016)	27959764	Case report	1	1	0	27
Scheibe F et al. (2017)	28003505	Case series	5	5	0	30·4
Boangher S et al. (2016)	28070431	Case report	1	1	0	66
Liao Z et al. (2017)	28070654	Case report	1	1	0	24
Patarata E et al. (2016)	28101036	Case report	1	1	0	36
Rypulak E et al. (2016)	28101360	Case report	1	1	0	23
Uchida Y et al. (2017)	28131220	Case report	1	1	0	20
Hattori Y et al. (2017)	28150403	Case report	1	1	0	35

Doden T et al. (2017)	28154283	Case report	1	1	0	24
Iglesias-Alonso A et al. (2017)	28186318	Case report	1	1	0	23
Fisher J et al. (2017)	28202296	Case report	1	1	0	27
Tsutsui K et al. (2017)	28223808	Case series	2	1	1	42
Lim EW et al. (2017)	28262407	Case report	1	0	1	65
Mugavin M et al. (2017)	28348631	Case report	1	1	0	52
Xiao X et al. (2017)	28418206	Case report	1	1	0	24
Kataoka H et al. (2017)	28445312	Case series	3	2	1	31
Mariotto S et al. (2017)	28478495	Case report	1	1	0	25
Han DY et al. (2017)	28503332	Case report	1	1	0	33
Chatterjee SS et al. (2017)	28558879	Case report	1	1	0	27
Herlopian A et al. (2016)	28616386	Case report	1	1	0	25
Dengler BA et al. (2017)	28644967	Case report	1	1	0	31
Strippel C et al. (2017)	28680648	Case report	1	1	0	67
Ding L et al. (2017)	28683723	Case report	1	0	1	55
Demma L et al. (2017)	28684142	Case report	1	1	0	28
Ueda A et al. (2017)	28780398	Case report	1	1	0	22
Van der Meulen AAE et al. (2017)	28822303	Case report	1	1	0	42
Li Y et al. (2017)	28824472	Case report	1	0	1	36
Rong X et al. (2017)	28859632	Case report	1	1	0	52
Ahmad J et al. (2017)	28884051	Case report	1	1	0	26
Sveinsson O et al. (2017)	28886955	Case report	1	1	0	26
Mutti C et al. (2017)	28905135	Case report	1	1	0	31
Jandu AS et al. (2016)	28979520	Case report	1	1	0	35
S Cahalan (2012)	n/a	Book	1	1	0	24

Supplementary table 3. Characteristics of main cohort and individual patient data subgroups. One-way ANOVA does not show statistically significant differences between these sub-groups using the above parameters and data (Individually-described All vs Group-described (P=0.964); Individually-described case reports vs. case series (P=0.894); Individually-described specialty journal sub-groups (P=0.429). HSVE = herpes simplex virus encephalitis.

	**Individually-described**			**Group-described**
	**All**	**Case reports**	**Case series**	
**Number of cases**	464	237/464 (51%)	226/464 (49%)	565
**Median age**	27	28	26	26
**Female n (%)**	368/464 (79%)	192/237 (81%)	176/226 (78%)	439/565 (78%)
**Psych n (%)**	439/464 (94%)	229/237 (96%)	210/226 (93%)	540/565 (96%)
**Seizure n (%)**	270/464 (58%)	143/237 (60%)	127/226 (56%)	381/565 (67%)
**Movement disorder n (%)**	256/464 (55%)	133/237 (56%)	123/226 (54%)	364/565 (64%)
**Dysautonomia n (%)**	209/464 (45%)	112/237 (47%)	97/226 (43%)	241/565 (43%)
**Central hypoventilation n (%)**	130/464 (28%)	79/237 (33%)	51/226 (23%)	189/565 (33%)
**Post-HSVE n (%)**	7/464 (1·5%)	7/237 (3%)	0/226 (0%)	0/565 (0%)
**Ovarian teratoma n (%)**	147/464 (32%)	82/237 (35%)	65/226 (29%)	209/565 (37%)

	**Individually-described**				
	**Psychiatry**	**Neurology**	**Internal medicine**	**Obstetrics & Gynaecology**	**Other**
**Number of cases**	78	249	66	17	54
**Median age**	27	27	28	27	26
**Female n (%)**	62/78 (79%)	191/249 (77%)	53/66 (80%)	17/17 (100%)	45/54 (83%)
**Psych n (%)**	78/78 (100%)	231/249 (86%)	62/66 (94%)	17/17 (100%)	51/54 (94%)
**Seizure n (%)**	43/78 (55%)	147/249 (59%)	39/66 (59%)	10/17 (58%)	31/54 (57%)

**Movement disorder n (%)**	40/78 (51%)	136/249 (55%)	47/66 (71%)	9/17 (53%)	24/54 (44%)
**Dysautonomia n (%)**	33/78 (42%)	103/249 (41%)	41/66 (62%)	10/17 (58%)	22/54 (41%)
**Central hypoventilation n (%)**	12/78 (15%)	72/249 (29%)	22/66 (33%)	6/17 (35%)	18/54 (33%)
**Post-HSVE n (%)**	0/78 (0%)	6/249 (2·4%)	1/66 (1·5%)	0/17 (0%)	0/54 (0%)
**Ovarian teratoma n (%)**	19/78 (24%)	65/249 (26%)	26/66 (39%)	12/17 (71%)	25/54 (46%)

Supplementary table 4. Comparisons of lower-level feature frequencies between aetiological and demographic sub-groups. P values shown for 2-way ANOVA with and without correction for multiple hypothesis testing.

**Comparison**	**P value**	**Bonferroni P**	**Benjamini-Hochberg P**
<40 vs. ≥40 years	0·902	1	0.962
Female vs. Male	0·649	1	0.962
Pregnancy-associated vs. Not	0·960	1	0.962
Ovarian teratoma vs. Not	0·962	1	0.962
Post-HSVE vs. Not	0·725	1	0.962
Isolated psychiatric presentation vs. Not	0·798	1	0.962

Supplementary table 5. Study cohort (n=464) sorted on proxy markers of psychiatric expertise. Cases in black boxes were categorised as 'psychiatric-described' (n = 78 + 51 = 129).

		**Psychiatry specialty journal**		
		-	+	*Row totals*
**Psychiatrist and/or Department of Psychiatry authorship**	+	**51**	**55**	106
	-	335	**23**	358
	Column totals	386	78	

Supplementary table 6. Network properties of non-psychiatric-described versus psychiatric-described sub-groups

Parameter	Non-psychiatric-described	Psychiatric-described
**Average degree**	21·481	27·259
**Average weighted degree**	208·444	394
**Graph density**	0·826	1·048
**Modularity**		
0.9 resolution threshold	0·086	0·046
0.8 resolution threshold	0·082	0·035
0.7 resolution threshold	0·077	0·029
**Average clusting coefficient**	0.886	0.975
**Average path length**	1.174	1.028
**Closeness centrality**		
Mean	0.905	0.979
Median	0.935	1
Range	0.655-1	0.85-1
**Betweenness centrality**		
Mean	2.36	0.416
Median	2.5	0.48
Range	0.1-5	0.05-0.48

Supplementary figure 1. A. The proportion of cases accounted for by different numbers of symptoms was assessed by stepwise addition of lower level psychiatric symptoms. B. The cohort was divided on the basis of being reported in a psychiatry journal and/or a psychiatrist and/or department of psychiatry on study authorship (‘psychiatric-described’, n=129; ‘non-psychiatric-described’, n=335). Each case is represented within a column and boxes are shaded if the lower level feature is present. The cases are sorted left to right by number of lower-level features within higher-level categories of descending frequency. C. Frequency of reported lower-level features sorted by specialty of journal in which they were reported. The proportion of features follows a similar pattern in all groups, however psychiatry-reported features in all domains are more frequently reported compared to the other specialties. D. Frequency distribution of number of lower-level features per patient sub-divided by specialty journal type.

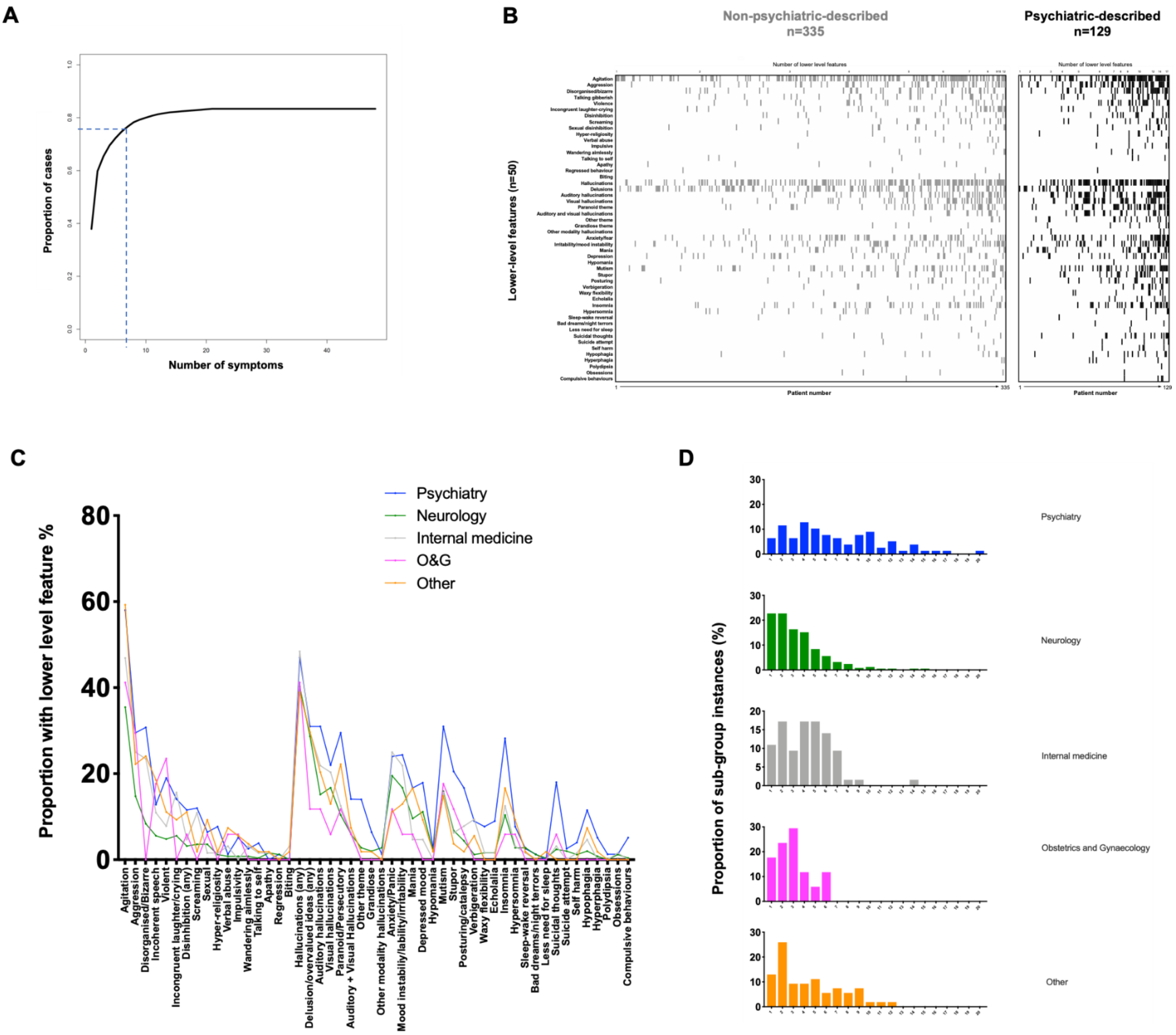

Supplement: Supplementary appendix [file mmc1.pdf]
